# Study on Salt-Frost Damage Durability of High-Performance Concrete with Polypropylene Fiber

**DOI:** 10.3390/ma18051007

**Published:** 2025-02-25

**Authors:** Zongao Qi, Yan Liu, Wei Zhang

**Affiliations:** College of Urban and Rural Construction, Hebei Agricultural University, Baoding 071001, China; 20222080166@pgs.hebau.edu.cn

**Keywords:** polypropylene fiber, high-performance concrete, flexural strength, salt freezing resistance, chloride ion penetration resistance

## Abstract

The durability of marine structures in the northern coastal areas is significantly damaged due to the double deterioration of chloride salt and freeze–thaw, and adding fiber can effectively improve the durability of marine structures. This work investigated the influence of polypropylene fiber content and salt freezing cycles on the flexural strength and durability of high-performance concrete through salt freezing cycle tests. The main experimental methods used included four-point load bending tests, relative dynamic elastic modulus tests, mass loss rate tests, and chloride ion permeability tests, with the mechanisms analyzed using SEM. The results indicated that an appropriate amount of polypropylene fibers significantly enhanced the flexural strength and durability of high-performance concrete. At a fiber content of 0.9 kg/m^3^, the concrete achieved the highest flexural strength. However, when the fiber content exceeded 0.9 kg/m^3^, excessive fibers caused uneven distribution and formed clusters, which reduced the flexural strength. At a fiber content of 1.2 kg/m^3^, the high-performance concrete showed optimal resistance to salt freezing and chloride ion permeability. However, exceeding this fiber content increased the concrete’s porosity, allowing harmful substances like chloride ions to penetrate more easily, thereby accelerating degradation under freeze–thaw conditions. This study contributes to a broader understanding of the durability of marine structures in coastal northern regions and provides theoretical data support for such environments.

## 1. Introduction

Marine structures in cold regions in winter will be subjected to the double deterioration of repeated freeze–thaw cycles and chloride ion erosion of seawater, which makes the actual service life of building structures far lower than the theoretical design value. Therefore, the durability of concrete structures under the double deterioration of chloride erosion and freeze–thaw cycles is more prominent, and the incorporation of fibers can effectively improve the durability of concrete structures. Compared with steel fiber, polypropylene fiber (PPF) is lightweight and has corrosion resistance, and it is cheaper than carbon fiber [1,2,3]. Therefore, polypropylene fiber is gradually applied to fiber-reinforced concrete.

At present, scholars have carried out a lot of experimental research and theoretical analysis on the mechanical properties and durability of polypropylene fiber concrete in terms of macroscopic and microscopic aspects [4,5]. Zhang et al. [6] tested the effects of different length-to-diameter ratios and contents of polypropylene fibers on the dynamic compressive properties of seawater sea sand recycled aggregate concrete and conducted an analysis using SEM. They found that, among the three strain rates, the sample with 9 mm long fibers at a content of 1.2 kg/m^3^ exhibited the highest peak stress. Letsosa et al. [7] tested the effects of different curing conditions on 5% synthetic polypropylene fiber-reinforced concrete and compared it with specimens without fibers. Ramírez et al. [8] tested the effects of different sizes of coarse aggregates on the mechanical properties and permeability of polypropylene fiber-reinforced pervious concrete specimens at two water-to-cement ratios (0.30 and 0.35). Studies [9,10,11] have shown that polypropylene fiber content, aspect ratio, shape, and other factors have different effects on the mechanical properties and durability of concrete. Akid et al. [12] tested the mechanical and durability properties of concrete with fly ash and polypropylene fibers. They found that replacing 15% by weight of cement with fly ash and adding 0.12% volume fraction of polypropylene fibers resulted in higher mechanical performance and durability for the concrete. Xu et al. [13] compared the effects of different polypropylene fiber dosages on peak stress and energy absorption capacity. They found that with the increase in polypropylene fiber content, both peak stress and energy absorption capacity first increased and then decreased, reaching their maximum values at a polypropylene fiber volume fraction of 0.6%. Li et al. [14] found that the shape and dosage of polypropylene fibers have an impact on the compressive strength of concrete. Concrete with a polypropylene fiber volume fraction of 0.90% exhibited the highest compressive strength. There is additional existing research [15,16,17] on the durability of polypropylene fiber concrete under water freezing, but the results do not agree because of the differences in the materials and methods of the tests. Zeng et al. [18] studied the effect of polypropylene fiber content on the permeability of concrete exposed to freeze–thaw cycles under compressive and tensile loads. Zhao et al. [19] tested the effects of different freeze–thaw mediums and their mass fractions on the freeze–thaw resistance of concrete. They found that the damage to concrete in a freeze–thaw environment with a salt solution as the medium was higher than that in a freeze–thaw environment with water as the medium. Lu et al. [20] tested the mechanical properties and pore structure deterioration of different types of fiber-reinforced concrete in five freeze–thaw mediums. They found that the porosity of the concrete specimens after freeze–thaw cycles was higher than that of the specimens in natural air. Li et al. [21] studied the effect of mineral admixtures and fibers on the freezing resistance of concrete. The environment of marine structures in cold regions is not a single freeze–thaw factor, and salt erosion also needs to be taken into account. A single freeze–thaw cycle test cannot fully study the durability of marine structures, so the study of the durability of polypropylene fiber-reinforced concrete structures under salt freezing coupling needs to be considered [22,23]. Huang et al. [24] tested the significant effects of basalt fibers, polypropylene fibers, and hybrid basalt-polypropylene fibers on the sulfate freeze–thaw erosion resistance of concrete. They found that the incorporation of fibers enhanced the concrete’s resistance to sulfate freeze–thaw erosion and inhibited the increase of pores and microcracks during the freeze–thaw erosion process. Ren et al. [25] proposed the “frost-salt heaving-corrosion” composite failure theory to explain the damage mechanism of concrete, considering water freezing, mirabilite precipitation, and sulfate corrosion. Gong et al. [26] tested the freeze resistance and salt freezing resistance of concrete with different dosages of polypropylene fibers. They found that the optimal freeze resistance and salt-frost resistance of polypropylene fiber concrete were achieved with a fiber dosage of 0.9 kg/m^3^.

Based on the above research, this experiment studied the mechanical properties and durability of polypropylene fiber high-performance concrete under salt freezing coupling and analyzed the influence of polypropylene fiber content and salt freezing cycles on high-performance concrete, which provided certain theoretical data support for the reliability of marine structures in complex environments.

## 2. Materials and Methods

### 2.1. Materials

P·O 42.5 ordinary Portland cement (with a compressive strength of no less than 42.5 MPa at 28 days, in accordance with the Chinese standard GB/T 17671-2021 [27]) was used in the test. The cement had a sieve residue of 10.9% on a 45 μm square hole sieve. The fly ash used was first-grade fly ash, with a sieve residue of 9.8% on a 45 μm square hole sieve. The silica fume was S95 grade, with a specific surface area of 19.1 m^2^/g. The fine aggregate consisted of river sand with a fineness modulus of 2.92, while the coarse aggregate was gravel with a continuous gradation of 5–25 mm, and the water-reducing agent was polycarboxylate superplasticizer. All raw materials were purchased from the Concrete Branch of Hebei Construction Group Co. (Baoding, China). The chemical composition of the cementitious materials was analyzed using X-ray fluorescence on an ARL PERFORM X4200W instrument, manufactured by Thermo Fisher Scientific (Waltham, MA, USA). The chemical composition of the cementitious material is shown in Table 1.

**Figure 1:**
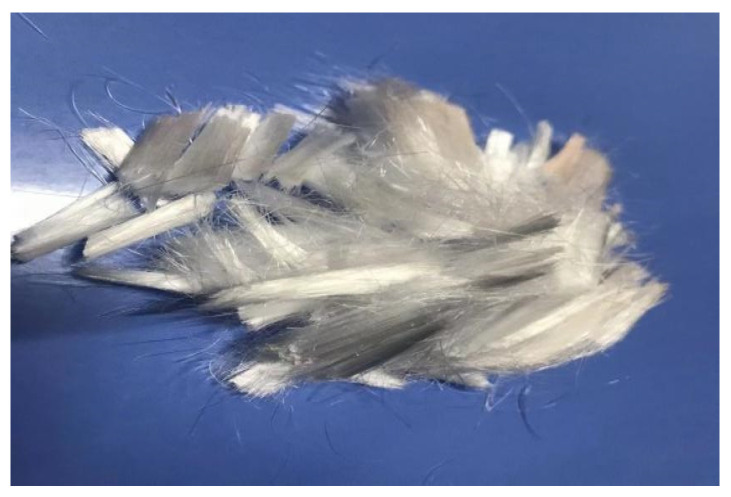
Polypropylene fiber.

The fibers were polypropylene fibers, each 19 mm in length, purchased from Laiyang Taichi Engineering Materials Co. (Laiyang, China), as shown in Figure 1.

In this experiment, the concrete strength grade C50 was designed according to the Chinese standard CECS 207-2006 [28] and the journal literature [29,30,31]. The design mix ratio was as follows: m(water):m(cement):m(fly ash):m(silica fume):m(sand):m(gravel):m(water reducing agent) = 185:320:155:40:615:1195:13.75. Based on this mix ratio, different amounts of polypropylene fiber 0.3, 0.6, 0.9, 1.2, and 1.5 kg/m^3^ were added, as shown in Table 2.

### 2.2. Methods

Polypropylene fiber high-performance concrete was prepared using the post-mixing method. The concrete raw materials (excluding the polypropylene fiber) were uniformly mixed using a forced mechanical mixer manufactured by China Academy of Building Research Co. (Beijing, China). Afterward, polypropylene fiber was gradually incorporated to prevent fiber agglomeration. The resulting concrete mixture was then placed into various specimen molds to create prisms with dimensions of 100 mm × 100 mm × 400 mm and cylindrical test blocks with a diameter of 100 mm and height of 50 mm. The concrete test blocks were molded for 1 day and then placed in a curing box for up to 28 days. After curing, the test blocks were removed and subjected to tests for flexural strength, frost resistance, chloride ion penetration resistance, and scanning electron microscopy analysis under different salt freezing cycles.

#### 2.2.1. Flexural Strength Test

**Figure 2:**
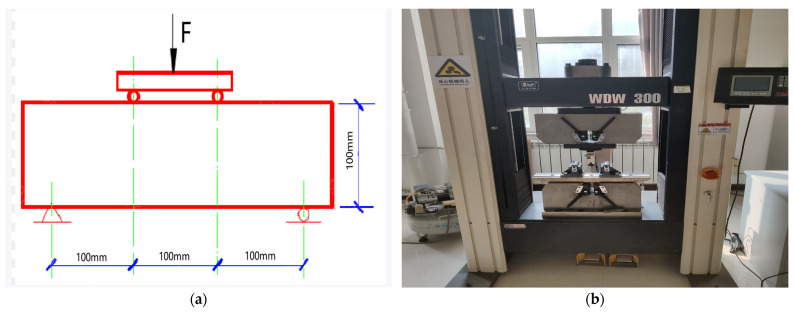
Flexural strength test: (**a**) flexural strength diagram, (**b**) WDW-300 universal testing machine.

Flexural strength was measured using the four-point load bending test method in accordance with the Chinese standard GB/T 50081-2019 [32]. The tests were conducted using the WDW-300 universal testing machine manufactured by Changchun Kexin Experimental Instrument Co. (Changchun, China), as shown in Figure 2. The specimen, with dimensions of 100 mm × 100 mm × 400 mm, was placed on the loading base of the test apparatus. The position was adjusted to ensure that the load was applied uniformly to the center of the specimen. The loading rate was 0.05 MPa/s, and the test data were recorded by the built-in acquisition system of the WDW-300 machine until the specimen failed.

#### 2.2.2. Freeze–Thaw Test

**Figure 3:**
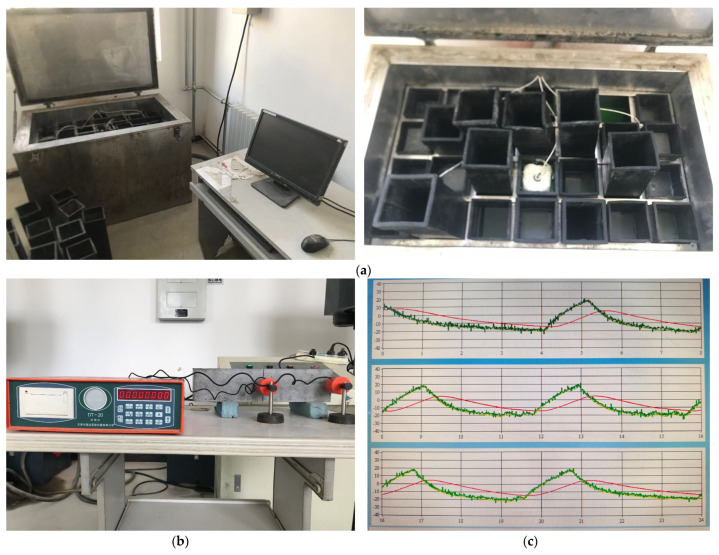
Freeze–thaw test: (**a**) freeze–thaw testing equipment, (**b**) DT-20 dynamic elastic modulus tester, (**c**) temperature variation curve.

The freeze–thaw test referred to the Chinese standards JTS/T 236-2019 [33] and GB/T 50082-2024 [34]. During the freeze–thaw cycles, each cycle was completed within 2 to 4 h. The minimum and maximum temperatures at the center of the specimen were maintained at (−18 ± 2 °C) and (5 ± 2 °C), respectively. The time for each specimen to decrease from 3 °C to −16 °C was not less than half of the freezing duration, and the time for each specimen to increase from −16 °C to 3 °C was not less than half of the total thawing duration. The cured test blocks were air-dried for two days and then immersed in a prepared 3.5% NaCl solution for two days and nights. After the test blocks were thoroughly soaked, the excess salt water on the surface was wiped off, and the blocks were then weighed and measured. Next, the test blocks were placed in the rubber barrel of the freeze–thaw box, and a 3.5% NaCl solution was added to begin the freeze–thaw cycle. After 25 freeze–thaw cycles, the test blocks were removed, the excess solution on the surface was wiped off, and their weight and dynamic elastic modulus were measured. The measured test blocks were reloaded into the rubber barrel, and the freeze–thaw cycles were repeated until the test met the required criteria. The freeze–thaw testing equipment, DT-20 dynamic elastic modulus tester, and temperature variation curve are shown in Figure 3.

#### 2.2.3. Chloride Ion Penetration Resistance Test

**Figure 4:**
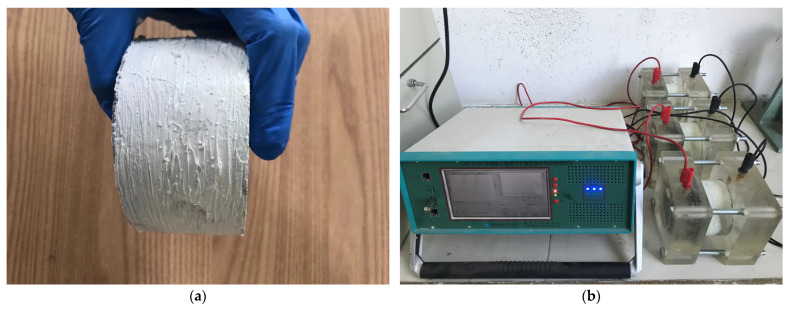
Electric flux test: (**a**) electric flux test block, (**b**) CABR-RCMP6 testing instrument.

The chloride ion penetration resistance test referred to the Chinese standards JTS/T 236-2019 [33] and GB/T 50082-2024 [34]. The chloride ion penetration resistance test adopted the electric flux method. The side of the cylinder test block with a diameter of 100 mm and a height of 50 mm was coated with a silica gel sealing material and placed in a saturator for vacuum saturation (dry pumping for 3 h, wet pumping for 1 h, soaking for 20 h). It was then placed in the fixture, and the CABR-RCMP6 testing instrument, manufactured by China Academy of Building Research Co. (Beijing, China), was used to conduct the electric flux test. The test specimen and testing equipment are shown in Figure 4.

#### 2.2.4. SEM Test

**Figure 5:**
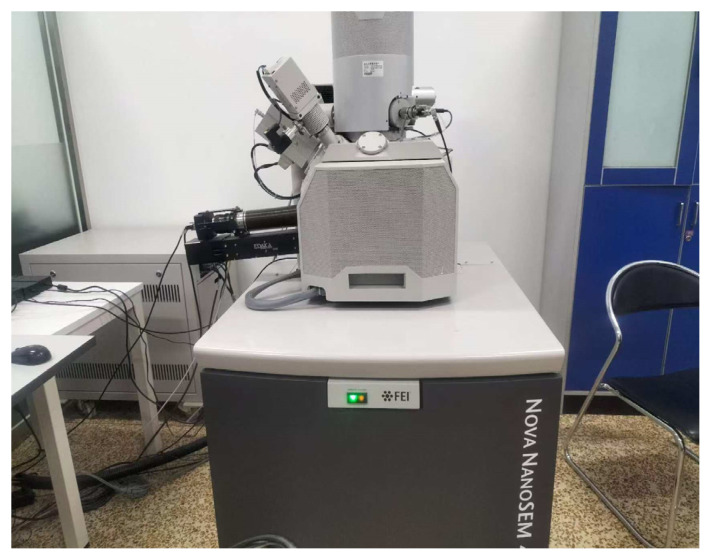
SEM testing equipment.

The test block was cut into 1 cm^2^ small pieces and soaked in anhydrous ethanol for 7 days. Subsequently, the pieces were placed in a drying oven at 60 °C for 24 h. After drying, they were placed in a vacuum gold plating instrument for gold plating on the surface of the samples and then transferred to the testing equipment for scanning electron microscope (SEM) analysis. The SEM instrument used was a Nova NanoSEM, manufactured by FEI Company (Hillsboro, OR, USA), as shown in Figure 5.

## 3. Results and Discussion

### 3.1. Mass Loss Rate

**Figure 6:**
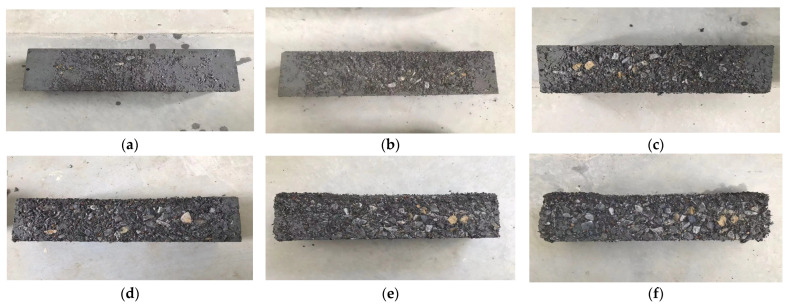
The salt freezing damaged test blocks of Group P1.2: (**a**) 25 cycles of salt freezing, (**b**) 50 cycles of salt freezing, (**c**) 75 cycles of salt freezing, (**d**) 100 cycles of salt freezing, (**e**) 125 cycles of salt freezing, (**f**) 150 cycles of salt freezing.

**Figure 7:**
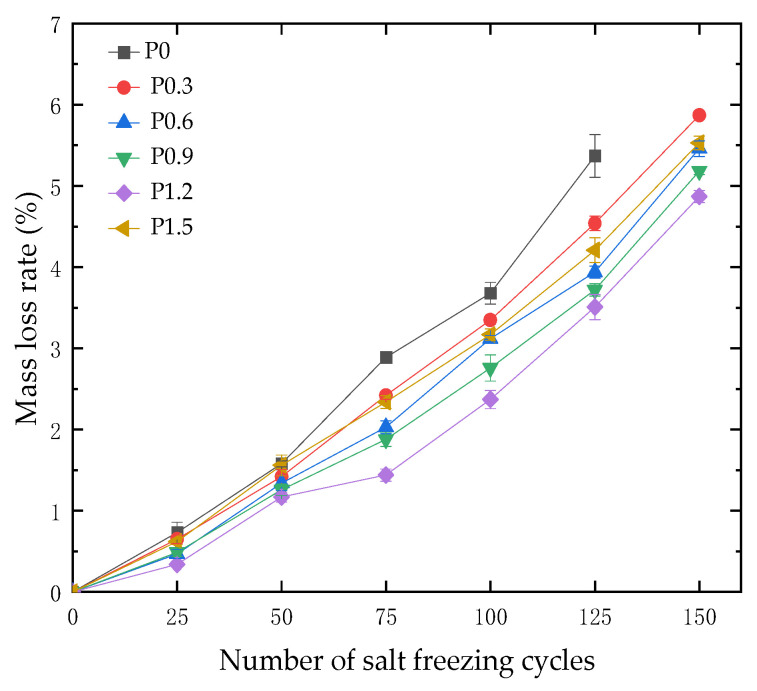
Effect of the number of salt freezing cycles on mass loss rate of concrete test specimens with different fiber contents.

After every 25 cycles of salt freezing coupling, the 100 mm × 100 mm × 400 mm test blocks were removed from the freeze–thaw box, the surface moisture of the test blocks was dried and weighed, and then the mass loss rate of the test blocks was calculated. The damage to the test blocks (taking Group P1.2 as an example) is shown in Figure 6, and the mass loss rate is shown in Figure 7.

As shown in Figure 7, before 50 cycles of salt freezing coupling, the mass loss rate of each group of high-performance concrete did not exceed 1.6%. When subjected to 75 cycles of salt freezing coupling, the differences in mass loss rates among the groups began to emerge. The P0 group exhibited the highest mass loss rate at 2.89%, while the P1.2 group had the lowest mass loss rate at 1.44%. After 125 cycles of salt freezing coupling, the mass loss rate of the P0 group was 5.37%, exceeding the 5% threshold considered as failure. After 150 cycles of salt freezing coupling, the mass loss rate of the P1.2 group was 4.87%, while the mass loss rates of other high-performance concrete groups exceeded 5%. From the above, it can be concluded that an appropriate amount of polypropylene fibers effectively reduced the mass loss rate of concrete under salt freezing cycles.

### 3.2. Relative Dynamic Elastic Modulus

**Figure 8:**
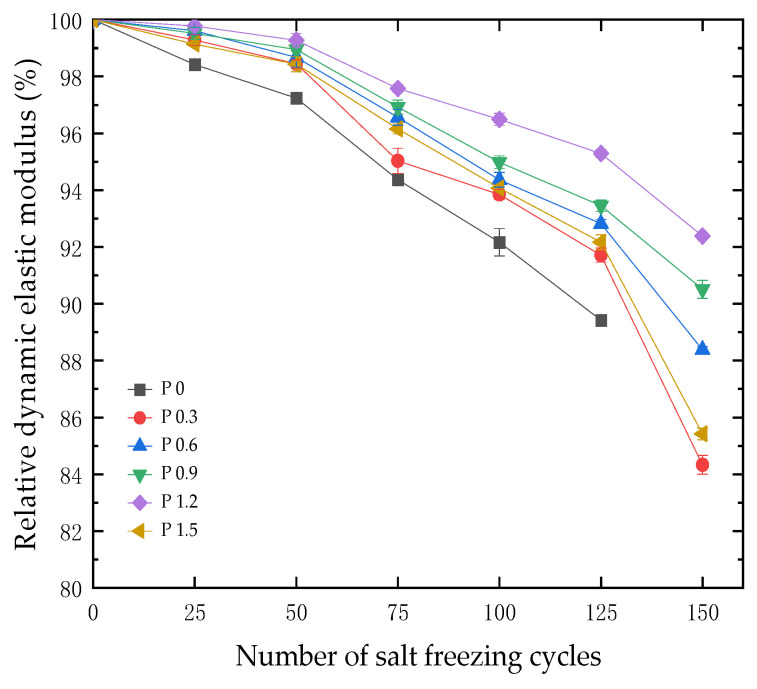
Effect of the number of salt freezing cycles on relative dynamic elastic modulus of concrete specimens with different fiber contents.

After 25 cycles of salt freezing coupling, the 100 mm × 100 mm × 400 mm test blocks were taken out of the freeze–thaw box, and the surface moisture of the test blocks was dried. The dynamic elastic modulus of the test blocks was tested using the dynamic elastic instrument, and then the relative dynamic elastic modulus was calculated, as shown in Figure 8.

It can be seen from Figure 8 that after 50 cycles of salt freezing, the relative dynamic elastic modulus loss of each group of test blocks with different fiber contents was smaller compared to the P0 group, with the P0 group at 97.24% and the other groups all above 98%. After 125 cycles of salt freezing, the relative dynamic elastic modulus of the P0 group was 89.42%. When the salt freezing coupling reached 150 cycles, the P0 group reached failure, while the relative dynamic elastic modulus of each group with other fiber contents remained above 84%, with the P1.2 group showing the best performance at 92.38%. When the polypropylene fiber content was lower than that of the P1.2 group, the relative dynamic elastic modulus loss rate of each test block gradually slowed as fiber content increased. However, when the fiber content exceeded that of the P1.2 group, the relative dynamic elastic modulus loss rate of the P1.5 group increased. From the above, it can be concluded that under the same number of salt freezing cycles, an appropriate amount of polypropylene fiber content slowed down the decrease in the relative dynamic elastic modulus of concrete.

### 3.3. Flexural Strength

**Figure 9:**
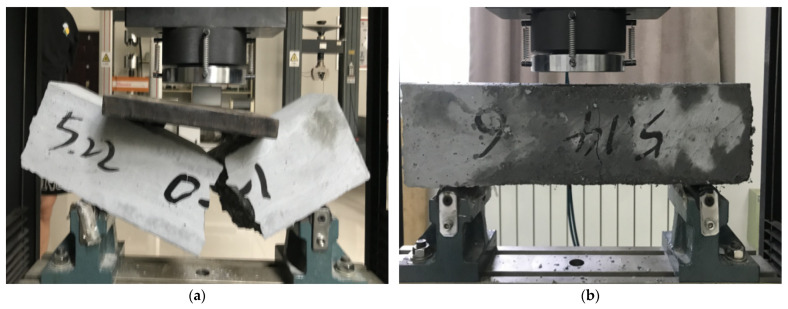
Effect of fiber content on the failure of the morphology of anti-fold test block: (**a**) P0 specimens, (**b**) P0.6 specimens.

The 100 mm × 100 mm × 400 mm test blocks under different salt freezing coupling cycles were placed on a universal testing machine for the flexural strength test, as shown in Figure 9.

During the testing process, for the specimens without fiber reinforcement, cracks appeared at the bottom of the weak area in the middle of the structure after reaching the ultimate load, and these cracks rapidly propagated to the top of the specimen, causing complete failure. For the fiber-reinforced specimens, cracks appeared at the bottom of the weak area in the middle of the structure after reaching the ultimate load, but they did not rapidly propagate through the specimen. The crack height reached approximately two-thirds of the specimen’s height, but the specimen did not completely fracture and still maintained a certain level of load-bearing capacity. Many fibers fractured and were pulled out between the cracks. The polypropylene fibers were randomly and disorderly distributed within the concrete matrix, and during loading, they acted as bridges, suppressing the development of microcracks and reducing the crack propagation rate in the specimens. After bending failure, the P0 group specimens fractured into two parts at the weak area in the middle of the structure, while for the P0.6 group, cracks appeared at the weak area in the middle of the structure after bending failure, and the constraining effect of the fibers helped keep the specimen together.

**Figure 10:**
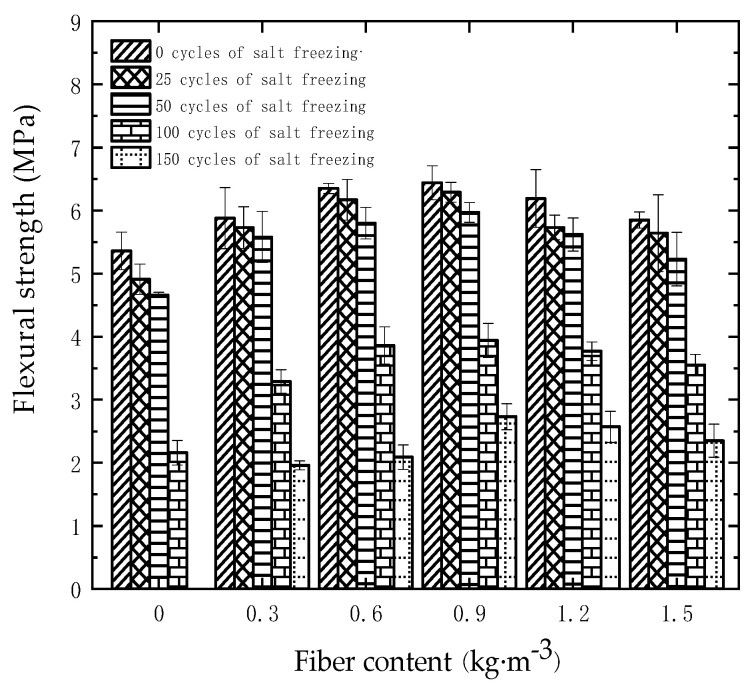
Effect of fiber content on flexural strength of concrete specimens under different numbers of salt freezing cycles.

Figure 10 illustrates the effect of fiber content on the flexural strength of concrete specimens under different salt freezing cycles. From the figure, it can be seen that before the salt freezing coupling, increasing the amount of polypropylene fibers had a positive impact on the flexural strength of high-performance concrete. The highest flexural strength of 6.44 MPa was achieved at a fiber dosage of 0.9 kg/m^3^. However, when the fiber dosage exceeded 0.9 kg/m^3^, the flexural strength of high-performance concrete decreased instead of increasing, with the P1.5 group showing a flexural strength of 5.85 MPa. Overall, the flexural strength of all fiber-reinforced groups was higher than that of the P0 group without fibers. After salt freezing coupling cycles, the freeze–thaw damage gradually reduced the flexural strength of the high-performance concrete specimens. When the salt freezing coupling reached 50 cycles, the flexural strength of all groups slightly decreased. Incorporating polypropylene fibers improved the salt freezing resistance of the high-performance concrete and suppressed crack propagation during bending failure. When the salt freezing coupling reached 100 cycles, the flexural strength of specimens with 0.9 kg/m^3^ fiber content was 3.94 MPa, which was 82% higher than the P0 group without fibers, and the freeze–thaw damage to all groups started to intensify. When the salt freezing coupling reached 150 cycles, the P0 group was damaged, as the mass loss rate of the specimens exceeded 5% after 125 salt freezing coupling cycles. The flexural strength of fiber-reinforced specimens was better than that of the P0 group, with the specimen containing 0.9 kg/m^3^ of fibers achieving the highest flexural strength of 2.73 MPa.

Under the effect of different numbers of salt freezing coupling cycles, when the fiber content increased from 0 to 0.9 kg/m^3^, the fibers tightly bonded with the matrix, forming a network structure that improved the internal pore structure of the high-performance concrete, enhancing the specimen’s toughness and flexural strength. However, when the fiber content exceeded 0.9 kg/m^3^, excessive fiber incorporation led to fiber clustering, reducing the bonding area between the mortar and aggregates, increasing the porosity within the concrete, and creating tiny pores and weak interfaces within the concrete matrix. This reduced the flexural strength of high-performance concrete, although the overall strength remained superior to that of the specimens without fibers.

### 3.4. Resistance to Chloride Ion Penetration

**Figure 11:**
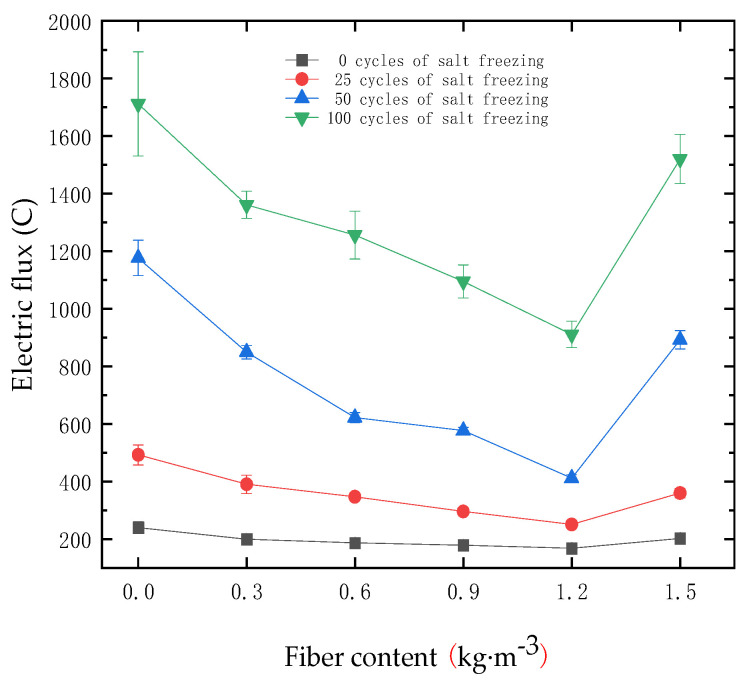
Effect of fiber content on the electric flux of concrete specimens under different numbers of salt freezing cycles.

The electric flux values of the test blocks with different salt freezing cycles and different fiber contents were measured by the CABR-RCMP6 testing instrument, as shown in Figure 11.

As shown in Figure 11, before the salt freezing coupling effect, the electrical flux values of the high-performance concrete specimens in each group initially decreased and then increased with the increase in polypropylene fiber content. When no fibers were added to the specimens, the electrical flux value was 239.803 C. As the fiber content was 1.2 kg/m^3^, the electrical flux reached the lowest value of 167.985 C, which was 29.95% lower than that of the specimens without fibers. As the fiber content continued to increase, the electrical flux value of the specimens with a fiber content of 1.5 kg/m^3^ increased to 202.252 C, though still lower than that of the specimens without fibers.

When the salt freezing coupling was 25 cycles, the electrical flux growth rate of each specimen was relatively slow. When the salt freezing coupling reached 50 cycles, the electrical flux growth rate of the specimens gradually accelerated. The electrical flux value of the specimens without fibers was 1176.409 C. After 100 salt freezing cycles, the electrical flux value of the specimen with 1.2 kg/m^3^ fiber content was 910.937 C, while the electrical flux values of the other groups were all higher than 1000 C. As the number of salt freezing cycles increased, the electrical flux value of each specimen gradually increased. The specimens without fibers showed the fastest increase in electric flux, while the specimens with 1.2 kg/m^3^ polypropylene fiber content exhibited the slowest increase in electric flux, outperforming the other fiber content specimens.

In summary, when the fiber content was less than 1.2 kg/m^3^, the chloride ion penetration resistance of the concrete increased with the increase in fiber content. However, when the fiber content exceeded 1.2 kg/m^3^, the chloride ion penetration resistance of the concrete decreased with the increase in fiber content. This is because an appropriate amount of fibers can be evenly distributed within the concrete, improving the internal channels and pore structure, thereby reducing the influence of external factors on the interior of the concrete. When the fiber content is too high, the fibers are unevenly distributed within the concrete, leading to clustering, which prevents the cementitious materials from bonding closely with the fibers. Additionally, excessive fibers cause the tiny pores in the concrete to connect through the fibers, which, under the continuous influence of deteriorating factors, reduces the internal density of the concrete and accelerates internal damage.

### 3.5. Microscopic Analysis

**Figure 12:**
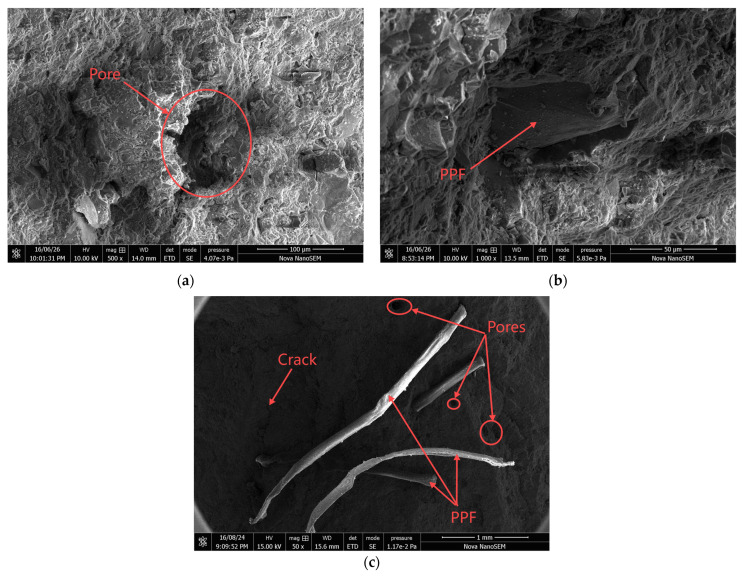
SEM images of polypropylene fiber high-performance concrete before salt freezing coupling: (**a**) P0 specimens, (**b**) P1.2 specimens, (**c**) P1.5 specimens.

**Figure 13:**
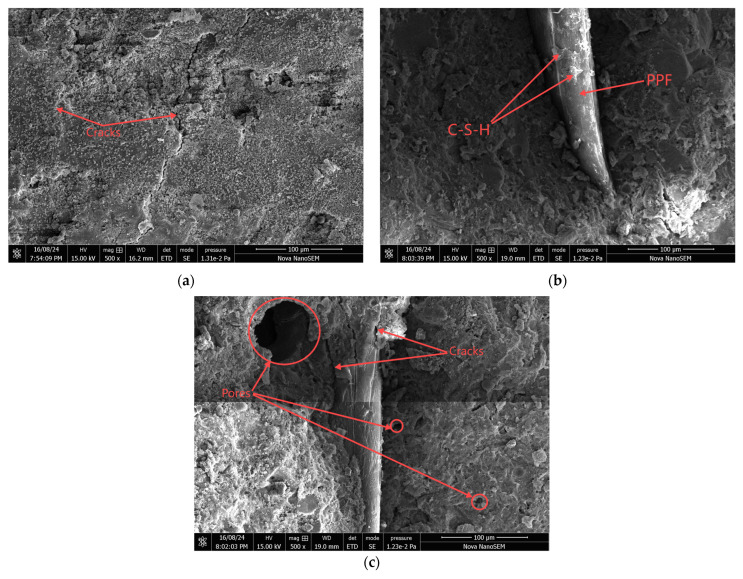
SEM images of polypropylene fiber high-performance concrete with 100 instances of salt freezing coupling: (**a**) P0 specimens, (**b**) P1.2 specimens, (**c**) P1.5 specimens.

The samples with different numbers of salt freeze–thaw cycles and fiber contents were analyzed using scanning electron microscopy (SEM), as shown in Figure 12 and Figure 13.

Figure 12 shows the SEM images of high-performance concrete with different polypropylene fiber content before salt freezing coupling. In Figure 12a, it can be observed that the P0 group sample, which did not contain polypropylene fibers, had larger internal pores and some small cracks around, with an overall lack of good compactness in the structure. In Figure 12b, it can be seen that the polypropylene fibers were closely bonded with the surrounding cementitious materials. This was mainly because the incorporation of polypropylene fibers inhibited the development of microcracks within the concrete, while the coarse aggregates altered the shape of the fibers, causing the fibers to interweave and improve the internal density of the concrete. In Figure 12c, the fiber distribution was dense, with many tiny pores and cracks around. The primary reason for this was that the excessive amount of polypropylene fibers in the preparation of high-performance formed fiber clusters and caused air to enter the concrete during the mixing process, resulting in increased internal porosity after the concrete had hardened.

Figure 13 shows the SEM images of polypropylene fiber high-performance concrete after 100 instances of salt freezing coupling. In Figure 13a, it can be observed that the P0 group exhibited an increasing number of cracks, which also widened. In Figure 13b, some C-S-H gel adhered to the fibers in the P1.2 group, and the fibers were still well bonded to the cement paste matrix. This was primarily because an appropriate amount of polypropylene fibers could improve the internal pore structure of the concrete, enhancing its freeze–thaw resistance and chloride ion permeability. In Figure 13c, compared to the P1.2 group, the P1.5 group showed many microcracks at the bond interface between the fibers and the cement matrix, with increasing internal porosity in the concrete matrix. This was due to the excessive fiber content in the specimens, which led to more internal air voids. Under the influence of solution osmotic pressure and thermal stresses during salt freezing coupling, these voids accelerated the internal erosion of the concrete matrix, increasing the tiny pores and cracks in the concrete.

## 4. Mechanism Analysis

Incorporating polypropylene fibers has positive and negative effects on the durability of high-performance concrete. On the macroscopic level, an appropriate amount of polypropylene fibers improves the ductility and flexural strength of the concrete, slows down the damage rate of concrete specimens under salt-freezing coupled deterioration, and enhances the chloride ion penetration resistance. However, excessive fiber incorporation reduces the flexural strength of the concrete and does not further improve the salt-freezing or ion penetration resistance. On the microscopic level, an appropriate amount of polypropylene fibers is evenly and randomly distributed throughout the concrete matrix, with the fibers tightly encapsulated by the cementitious material. This helps inhibit the development of tiny cracks and voids, thereby improving the overall density of the concrete. On the other hand, excessive fiber incorporation during the preparation process causes the fibers to cluster together, introducing more air into the concrete and increasing the internal structure’s porosity. Furthermore, the bond between some cementitious materials and fibers weakens, forming small cracks. Erosion from external unfavorable factors can penetrate the concrete through these tiny pores, accelerating the damage.

Similarly, the chloride salt solution has two effects on high-performance concrete during the freeze–thaw process. The beneficial effect is that, during the early stages of the salt freezing coupling cycle, chloride salts penetrate the concrete specimen and react to form chloride salt hydrates and chloride salt crystals, thereby enhancing the compactness of the concrete. Additionally, the freezing point of sodium chloride solution is lower than that of pure water, which benefits the early-stage freeze resistance and impermeability of the concrete. Therefore, before reaching 50 cycles of salt freezing coupling, the relative dynamic modulus of elasticity and mass loss rate of the specimen remains relatively unchanged. The detrimental effect is that, with the increase in the number of salt freezing coupling cycles, the chloride salt hydrates and chloride crystals formed inside the concrete continue to expand, accelerating internal damage to the concrete. Moreover, the osmotic pressure of the chloride salt solution is higher than that of pure water, which results in a concentration gradient between the surface and the internal environment of the concrete, creating greater osmotic pressure. The concrete is also subjected to fatigue stress due to the combined effects of salt and freeze–thaw cycles. This coupling of thermal cycling-induced stresses and osmotic pressure of the salt solution accelerates the development of microcracks and pores inside the concrete matrix, as well as the spalling and erosion of the external cement mortar.

## 5. Conclusions

Based on experimental research and theoretical analysis, the following results and conclusions are obtained:

(1) The flexural strength of the concrete was effectively improved when the fiber content increased from 0 to 0.9 kg/m^3^. This improvement is due to the network structure formed by the fibers in the concrete, which tightly integrates with the cementitious materials, enhancing the ductility and flexural strength of the concrete. However, when the fiber content exceeds 0.9 kg/m^3^, the flexural strength of the concrete decreases. This is because the excessive fiber content leads to uneven fiber distribution, forming clusters, and introduces more air during the concrete preparation process, which increases the porosity of the concrete and negatively affects its flexural strength.
(2) The incorporation of polypropylene fibers effectively improves the salt-freezing resistance of concrete. Compared to the P0 group, after 100 salt freezing cycles, the relative dynamic modulus of elasticity of the P1.2 group concrete specimens increased by 4.7%, while the mass loss rate and electrical flux decreased by 35.6% and 46.78%, respectively. The random distribution of fibers forms a network structure that blocks the connection of tiny pores within the concrete, inhibiting the development of microcracks caused by salt freezing damage. However, when the fiber content exceeds 1.2 kg/m^3^, the excessive amount of fibers increases the porosity of the concrete, making it easier for harmful substances, such as chloride ions, to penetrate, which accelerates deterioration under freeze–thaw conditions.
(3) In the early stages of salt freezing, chloride hydrates and chloride salt crystals enter the high-performance concrete, effectively improving the internal density of the concrete and slowing the rate of salt freezing damage. However, as the number of salt freezing cycles increases, the chloride hydrates and chloride salt crystals within the high-performance concrete continue to expand, generating crystallization pressure, which accelerates the internal damage of the concrete.
(4) The durability damage of polypropylene fiber high-performance concrete gradually increases with the number of salt freezing cycles. The appropriate amount of fiber incorporation improves the durability of the concrete. However, when the fiber content exceeds 1.2 kg/m^3^, as the number of salt freezing cycles increases, the internal porosity of the concrete increases. The continuous detachment of the cement mortar and fibers from the concrete surface allows harmful agents to penetrate more easily. Additionally, temperature stress, osmotic pressure, and crystallization pressure exacerbate the internal damage of the concrete, leading to a continuous decline in the durability of the polypropylene fiber high-performance concrete.

## Figures and Tables

**Table 1:** Chemical composition of cementitious materials.

Cementitious Materials	Chemical Composition Ratio/%						
	SiO_2_	Al_2_O_3_	Fe_2_O_3_	CaO	K_2_O	TiO_2_	Cl^−^
Cement	17.32	4.04	3.32	59.74	0.90	0.30	0.03
Fly ash	49.30	28.37	5.81	3.08	2.67	1.28	0.12
Silica fume	96.31	0.19	0.12	0.12	0.01	0.02	0.07

**Table 2:** Mix ratio of high-performance concrete.

Sample	Mix Ratio/(kg·m^−3^)							
	Water	Cement	Fly Ash	Silica Fume	Sand	Gravel	Water Reducing Agent	Polypropylene Fiber
P0	185	320	155	40	615	1195	13.75	0
P0.3	185	320	155	40	615	1195	13.75	0.3
P0.6	185	320	155	40	615	1195	13.75	0.6
P0.9	185	320	155	40	615	1195	13.75	0.9
P1.2	185	320	155	40	615	1195	13.75	1.2
P1.5	185	320	155	40	615	1195	13.75	1.5

## Data Availability

The original contributions presented in this study are included in the article. Further inquiries can be directed to the corresponding authors.
